# Validating Prediction Tools for Autoimmune Encephalitis in Adult Taiwanese Patients: A Retrospective Study

**DOI:** 10.3390/biomedicines11071906

**Published:** 2023-07-05

**Authors:** Yan-Ting Lu, Chih-Hsiang Lin, Chen-Jui Ho, Shih-Ying Chen, Meng-Han Tsai

**Affiliations:** 1 Department of Neurology, Kaohsiung Chang Gung Memorial Hospital, College of Medicine, Chang Gung University, Kaohsiung 833401, Taiwan; yantimlu@yahoo.com.tw (Y.-T.L.);; 2 College of Medicine, Medical School, Chang Gung University, Taoyuan 333323, Taiwan; 3 Genomics and Proteomics Core Laboratory, Department of Medical Research, Kaohsiung Chang Gung Memorial Hospital, Kaohsiung 833401, Taiwan

**Keywords:** autoimmune encephalitis, antibody prevalence in epilepsy score, response to immunotherapy in epilepsy score, anti-NMDAR encephalitis one-year functional status score, validation, autoimmune diseases of the nervous system, anti-N-methyl-D-aspartate receptor encephalitis

## Abstract

Autoimmune encephalitis (AE) is a neurological emergency. We aimed to analyze the application and effectiveness of the currently available prediction tools for AE patients in Taiwan. We retrospectively collected 27 AE patients between January 2008 and December 2019. Antibody Prevalence in Epilepsy (APE) score, Response to Immunotherapy in Epilepsy (RITE) score, and anti-NMDAR Encephalitis One Year Functional Status (NEOS) score were applied to validate their usability. Based on the defined cutoff values, the sensitivity and specificity of each score were calculated. A receiver operating characteristic (ROC) curve and the area under the curve (AUC) were generated for each scoring system. The AUC value of APE was 0.571. The AUC value of RITE was 0.550. The AUC values for the NEOS score at discharge and long-term follow-up were 0.645 and 0.796, respectively. The performance of APE and RITE scores was suboptimal in the Taiwanese cohort, probably due to the limitations of the small sample size and single ethnicity. On the other hand, the NEOS score performed better on long-term follow-up than at discharge.

## 1. Introduction

Autoimmune encephalitis (AE) is a rare and debilitating but potentially treatable neurological emergency [1]. AE is caused by the production of autoantibodies against the neuron-surface or synaptic proteins [2], and patients often present acutely with severe memory deficits, changes of consciousness, behavior changes, psychiatric symptoms, cognitive impairment, and seizures [3]. AE is also increasingly recognized as an etiology of status epilepticus (SE) and non-convulsive SE [4,5]. Despite the severe initial symptoms, favorable long-term outcomes can be achieved with early diagnosis, appropriate and aggressive immunotherapy, and/or tumor removal [6]. During the treatment course, the patient may receive various immunotherapies, including steroid pulse therapy, intravenous immune globulin (IVIG), plasma exchange, or monoclonal antibodies. Currently, there is no consensus guideline regarding how the various immunotherapies should be used, and most of the treatment plans are empirical. In clinical practice, the diagnosis of AE is not straightforward, and it may be difficult to differentiate from infectious encephalitis such as viral encephalitis of enteroviral origin, rabies, and herpes simplex-1 (HSV-1) [5]. HSV encephalitis is one of the viral infections that may resemble AE, which shares similar presentations such as altered consciousness level, new-onset seizure, and behavior change [5]. Both the diagnosis and treatment of AE were challenging. Objective evaluation methods can facilitate the correct diagnosis and guide the treatment strategy. Currently, three scoring systems are used to predict the different aspects of AE. The Antibody Prevalence in Epilepsy (APE) score [7] was developed to predict the presence of antibodies in patients with epilepsy of unknown etiology. The Response to Immunotherapy in Epilepsy (RITE) score [8] is used to estimate the response to immunotherapy, and the Anti-NMDAR Encephalitis One-Year Functional Status (NEOS) score [9] focuses on predicting the functional recovery of patients with anti-N-methyl-D-aspartate receptor (NMDAR) encephalitis.

Only a few studies have applied these prediction models to AE patients. One Chinese study used the APE^2^ score to predict the presence of autoantibodies in patients with AE, and the result showed high sensitivity and specificity [10]. The study recruited 180 inpatients with a diagnosis of possible AE, among whom 32 had AE-related antibodies in serum and/or cerebrospinal fluid (CSF), and the detection rate of AE-related antibodies was 17.8%. With a cutoff score of five, the APE^2^ score showed high sensitivity (0.875) and specificity (0.791).

In Taiwan, the diagnosis of AE is aided by autoantibody testing. However, the diagnostic cell-based autoantibodies test is neither available everywhere nor covered by the National Health Insurance program in Taiwan. An antibody prediction mode may help the diagnosis of AE, and the treating physician may initiate immunotherapy as soon as possible or search for an alternative diagnosis. A disease severity prediction mode may assist in the decision-making process about the aggressiveness of immunotherapy to prevent delayed treatment. The aim of the present study is to evaluate the effectiveness of the currently available scoring systems related to AE and assess whether they can be applied to Taiwanese patients with AE. In this study, we investigated possible AE, which includes AE associated with neuronal surface antibodies and “seronegative” AE.

## 2. Methods

### 2.1. Study Design and Patients

In this retrospective study, we enrolled patients older than 20 years old diagnosed with AE at Kaohsiung Chang Gung Memorial Hospital, Taiwan, between January 2008 and December 2019. Kaohsiung Chang Gung Memorial Hospital is a tertiary center in southern Taiwan. We enrolled patients who fulfilled the criteria for possible AE according to expert consensus [3]. Patients with systemic autoimmune diseases with CNS manifestations were excluded. The clinical data were obtained by retrospectively reviewing the digital medical record. The enrolled patients were evaluated by neurologists for the following scoring systems and functional outcome status. This study was approved by the Institutional Review Board of Kaohsiung Chang Gung Memorial Hospital.

### 2.2. Definitions and Criteria

Patients with AE were diagnosed as suggested by previous expert consensus criteria [3], as shown in Table 1:

Patients who fulfilled these criteria were further tested with a commercial cell-based assay (CBA) for AE (Autoimmune Encephalitis Mosaic 6 provided by EUROIMMUN), which included anti-NMDAR, anti-CASPR2, anti-AMPAR, anti-LGI1, anti-DPPX, and anti-GABABR. The assay was to test for known autoantibodies in patients’ blood and/or CSF samples. Patients with clinical suspicion of AE but who test negative with the commercial CBA will be considered to have AE based on their response to treatment. Response to immunotherapy or tumor treatment will be required for enrollment in the study and classified as seronegative AE. All patients underwent routine clinical surveys, including blood biochemistry, CSF, electroencephalography, and brain MRI, to rule out other similar conditions that may mimic AE, such as viral encephalitis of enteroviral origin, rabies, varicella-zoster virus, and herpes simplex-1 (HSV-1). Other autoimmune disease markers, including anti-nuclear antibodies, anti-dsDNA, anti-extra nuclear antigens, celiac disease, and anti-thyroid autoantibodies, were also tested. Systemic autoimmune disease-related encephalitis such as lupus-related encephalitis, Sjögren’s syndrome, primary or secondary angiitis of the central nervous system, or Hashimoto’s encephalitis, were excluded.

### 2.3. The Antibody Prevalence in Epilepsy (APE) Score

The Antibody Prevalence in Epilepsy (APE) [7] score was designed to predict the presence of neural antibodies in AE patients according to the initial clinical features and surveys. The clinical features include mental status change, neuropsychiatric symptoms, autonomic dysfunction, viral prodrome, facial dyskinesia, and responsiveness of seizures to anti-seizure medications (ASMs), and the clinical surveys include CSF findings, MRI presentations, and underlying malignancy. An APE score ≥ 4 indicates a high probability of the presence of neural antibodies. Our AE patients received commercially available cell-based neuronal surface autoantibody testing (EUROIMMUN, Autoimmune Encephalitis Mosaic 6 assay, Germany) to test for currently known autoantibodies, including NMDAR, LGI1, CASPR2, AMPAR, and GABAR. Those who did not receive testing were not evaluated by the APE score.

### 2.4. The Response to Immunotherapy in Epilepsy (RITE) Score

The Response to Immunotherapy in Epilepsy (RITE) score was developed to predict the response to immunotherapy [8]. The RITE score includes all 10 items of the APE score and two additional items: (1) immunotherapy initiated within six months of symptom onset and (2) detection of neural plasma membrane autoantibody.

The RITE score ≥ 7 predicts a favorable functional outcome following immunotherapy for AE. A favorable functional outcome was defined as ≥1 change in the modified Rankin Scale (mRS) score at discharge compared with that at admission. First-line immunotherapy includes intravenous high-dose steroids (methylprednisolone), intravenous immunoglobulin (IVIG), and/or plasmapheresis. Second-line immunotherapy includes rituximab and/or cyclophosphamide [11].

### 2.5. Anti-NMDAR Encephalitis One Year Functional Status (NEOS) Score

The NEOS score is a 5-point prediction score including: (1) intensive care unit (ICU) admission required; (2) no clinical improvement after 4 weeks of treatment; (3) no treatment within 4 weeks of symptom onset; (4) abnormal MRI findings; and (5) CSF WBC count > 20 cells/μL. The NEOS score focuses on predicting the functional recovery of patients with anti-NMDAR encephalitis, with a higher score indicating the probability of poor functional status at 1 year [9]. The total score ranges from 0 to 5, and we used a cutoff point of 3 to predict the functional outcome of our patients in this study. We applied the prediction model to our patients with anti-NMDAR encephalitis. To validate if NEOS can also be applied to AE patients without anti-NMDAR antibodies, we did another analysis that applied NEOS to all patients in our cohort, regardless of the etiology of AE. A good outcome was defined as an mRS score ≤ 2. Two time points were evaluated for the outcome: the first was at discharge, and the second was 1 year after discharge. If the patient was lost to follow-up within 1 year, the mRS score at the last visit was used.

### 2.6. Statistical Analysis

Statistical analyses were performed using SPSS for Windows (version 20; IBM Corp., Armonk, NY, USA). Based on the designated cutoff value of each scoring system, sensitivity and specificity were calculated. A receiver operating characteristic (ROC) curve was generated for each scoring system. Fisher’s exact test was used to compare the clinical features between survivors and non-survivors. We applied linear regression analysis to find possible confounding factors in our cohort, such as the effect of age and sex on the three scores. Categorical variables were assessed using Fisher’s exact tests, and continuous variables were compared using the Mann–Whitney U-test. *p* < 0.05 was considered statistically significant. The Shapiro–Wilk normality test was used to check the normal distribution of continuous variables.

## 3. Results

### 3.1. Patient Profile

During the 12 year study period, 27 patients diagnosed with AE were enrolled, including twenty-one females and six males. The median age at diagnosis was 26 years (interquartile range [IQR] = 21–34.5 years). The median duration of hospitalization was 47 days (IQR = 31.5–69.5). Twenty-six (96.3%) patients were admitted to the intensive care unit, where the median stay was 35 days (IQR = 15.5–55). The Shapiro–Wilk normality test revealed the distribution of age and days of ICU stay was not normally distributed, while the duration of hospitalization followed a normal distribution. The linear regression analysis revealed that neither age nor sex influenced the outcomes of the three scores. Twenty-six (96.3%) patients had an APE score ≥4, 23 of the 24 patients with immunotherapy (95.8%) had a RITE score ≥ 7, and only two (7.4%) patients had a NEOS score ≥ 4. Nineteen patients (70.3%) had an mRS score ≤ 2 at discharge, and 20 (95.2%) had an mRS score ≤ 2 at 1 year or the last visit. Five (18.5%) patients died, and one was lost to follow-up.

Six patients did not receive the limbic encephalitis panel examinations. All patients were tested for the other markers of autoimmune disease, and five were positive for anti-ENA antibodies, five were positive for anti-thyroglobulin antibodies, four were positive for anti-TPO antibodies, three were positive for anti-SSA, one was positive for anti-dsDNA, and one was positive for ANA. Among the 21 patients who received the limbic encephalitis panel, ten tested for both CSF and serum, and the other 11 only tested for CSF. Among the ten patients who tested for both CSF and serum, five were positive for anti-NMDAR antibodies in CSF only. The remaining five had negative results in both CSF and serum. Among the 11 patients who tested for CSF only, nine were positive for anti-NMDAR antibodies. Twenty-five (92.6%) patients had new-onset epileptic seizures, and one patient had an epilepsy history. One male patient with the diagnosis of anti-NMDAR encephalitis did not have an epilepsy history but had two seizure episodes 4 months before AE. Nineteen patients had de novo SE. The onset symptoms included consciousness disturbance (*n* = 8), seizure (*n* = 5), psychiatric change (*n* = 5), viral prodrome (*n* = 4), fever (*n* = 2), speech disturbance (*n* = 2), and memory decline (*n* = 1). Seventeen patients had psychiatric symptoms, including bizarre behavior in seven, mood instability in four, hallucinations in three, anxiety in one, delusion in one, and psychosis in one.

Four female patients had AE associated with pregnancy: two with de novo SE during pregnancy and two with de novo SE during the postpartum period. Four females with anti-NMDAR encephalitis had ovarian teratoma (4/11, 36.3%), three of whom underwent removal of the teratoma. Detailed demographic data on the enrolled patients are presented in Table 2.

### 3.2. Treatment and Prognosis

Among the 14 anti-NMDAR encephalitis patients, eleven started the immunotherapy within 4 weeks of onset, two after 4 weeks, and one did not receive any immunotherapy. Among the 11 patients who received treatment within 4 weeks, six received first-line treatment only, and five received both first- and second-line treatment. For the two patients who received immunotherapy over 4 weeks after disease onset, one received first- and second-line treatment simultaneously on the same day, and the other had an interval of 2 weeks. The patient who did not receive immunotherapy improved clinically after only receiving ASMs. The first-line immunotherapy for the 13 patients included six steroid pulse therapies, five steroid pulse therapies plus plasmapheresis, and two steroid pulse therapies plus IVIG. One patient received up to four courses of plasmapheresis. Both patients with IVIG received a single course.

Of the 13 patients with no known autoantibodies or who did not receive limbic encephalitis panel examinations, nine had first-line immunotherapy only, two received first- and second-line immunotherapy within a 2 week interval, and two only received low-dose steroids. The two patients who only received low-dose steroids had other comorbidities. One was a female patient diagnosed with AE during pregnancy who received a Cesarean section with low-dose steroids to prevent infantile pulmonary immaturity. The other only received low-dose steroids for AE because of concurrent sepsis during treatment for AE. The patient who only received low-dose steroids died, and the pregnant AE patient had seizure control after the Cesarean section.

The overall mortality rate of our patient cohort at discharge was 18.5% (5/27). No patients died in the anti-NMDAR encephalitis group. All the patients who died were female, two of whom did not receive limbic encephalitis panel examinations, and the other three were seronegative for AE. The clinical characteristics of the survivors and non-survivors are shown in Table 3. Psychiatric symptoms were the most prominent major clinical symptom in the survivors (*p* = 0.0473). There were no significant differences between the two groups in SE, the need for an ICU stay, or abnormal MRI findings. The incidence of elevated CSF protein (protein ≥ 50) was higher in the non-survivor group (*p* = 0.0016), but there were no obvious differences in CSF WBC count or IgG index between the two groups (three patients had no IgG index data, two in the survivor group and one in the non-survivor group). The mortality rate was lower in the patients with anti-NMDAR encephalitis compared with the autoantibody-negative group (*p* = 0.0263); however, there was no sex difference (*p* = 0.5552).

### 3.3. Antibody Prevalence in Epilepsy (APE) Score and Antibody Prediction

**Figure 1:**
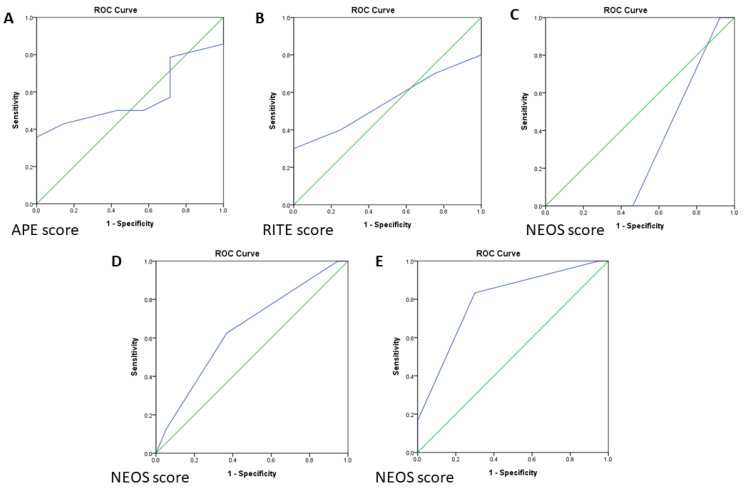
(**A**) ROC (Receiver operating characteristic curve) analysis of the APE score for autoantibody prediction. (**B**) ROC analysis of the predictive value of the RITE score for favorable immunotherapy response. (**C**) ROC analysis of the NEOS score in anti-NMDAR encephalitis at discharge. (**D**) ROC analysis of the NEOS score for all AE patients at discharge. (**E**) ROC analysis of the NEOS at 1 year follow-up/long-term follow-up for all 26 patients except one lost to follow-up. ROC, receiver operating characteristic.

Twenty-one patients received the limbic encephalitis panel examination, of whom fourteen (66.7%) had anti-NMDAR encephalitis and seven (33.3%) were negative for the known autoantibodies. A cutoff APE score of ≥ 4 was used to suggest the presence of neural antibodies, twenty (95.2%) patients had an APE score ≥ 4, and one (4.8%) had an APE score < 4. The sensitivity and specificity of the APE score were 92.9% and 0%, respectively. ROC curve analysis of the APE score showed an area under the curve (AUC) value of 0.571 (95%, 0.325–0.817) (Figure 1A).

### 3.4. RITE Score for Response to Immunotherapy

In the RITE score analysis, we excluded three patients who did not use steroids or only received low-dose steroids. Of the remaining 24 patients, 23 had a RITE score ≥ 7, and 19 (82.6%) of these 23 patients had a favorable outcome (mRS score improvement ≥ 1 at discharge). Only one patient had a RITE score < 7, but they still had a favorable outcome. The median RITE score was 11 (IQR = 9–14.5). The sensitivity and specificity for a favorable immunotherapy response were 95% and 0%, respectively, and the AUC value was 0.550 (95% CI, 0.312–0.788) (Figure 1B).

We performed a subgroup analysis that only included patients who received panel tests for AE. All 13 patients with anti-NMDAR encephalitis who received immunotherapy (*n* = 13, excluding the one only with ASMs) had a RITE score ≥ 7, and all had a favorable outcome. The ROC curve of RITE could not be estimated as all 13 patients improved clinically. The autoantibody-negative group included six patients (one with low-dose steroids was excluded). Five of these patients had a RITE score ≥ 7, of whom three had a favorable outcome. The patient with a RITE score < 7 also had a favorable outcome. The sensitivity and specificity for a favorable immunotherapy response were 75% and 0%, respectively, and the AUC value was 0.438 (95% CI, 0–0.913).

### 3.5. Outcome Prediction Score at Discharge and 1 Year Follow-Up

Among the 14 patients with anti-NMDAR encephalitis, one (7.1%) had a NEOS score of 0, seven (50%) had a score of 2, five (35.7%) had a score of 3, and one (7.1%) had a score of 4. At discharge, only one patient (7.1%) had an mRS score of 3, and the other thirteen patients had an mRS score ≤ 2. All patients had an mRS score ≤ 2 at 1 year follow-up or in long-term follow-up time, except for one patient who was lost to follow-up. With 3 as the cutoff NEOS score, the sensitivity and specificity of the NEOS score to predict functional outcome at discharge were 0% and 53.8%, respectively. The sensitivity of the NEOS score to predict functional outcome at 1 year follow-up or in long-term follow-up time could not be estimated because all patients had a good outcome and the specificity was 61.5%. The AUC value of the NEOS score in the anti-NMDAR encephalitis patients at discharge was 0.308 (95% CI, 0.000–0.663); however, the AUC value for 1 year follow-up or in long-term follow-up could not be estimated as all prognoses were good. The ROC curve of the NEOS score in the anti-NMDAR encephalitis patients at discharge is shown in Figure 1C.

When the NEOS score was applied to all 27 AE patients, one patient (3.7%) had a NEOS score of 0, fourteen (51.9%) had a score of 2, ten (37%) had a score of 3, and two (7.4%) had a score of 4. Eight patients (29.6%) had an mRS score > 2 at discharge, and the remaining patients had an mRS score ≤ 2. Only one patient (4.8%) had an mRS score > 2 at 1 year follow-up or in long-term follow-up. Five patients died, and one was lost to follow-up. With 3 as the cutoff NEOS score, the sensitivity and specificity to predict the outcome at discharge were 62.5% and 63.2%, respectively. The sensitivity and specificity to predict the outcome at 1 year follow-up or in long-term follow-up were 83.3% and 70%, respectively. The ROC curves of the NEOS score at discharge, 1 year follow-up, or long-term follow-up are presented in Figure 1D. The AUC values for NEOS score at discharge and 1 year follow-up or in long-term follow-up were 0.645 (95% CI, 0.415–0.874) and 0.796 (95% CI, 0.588–1), respectively (Figure 1E).

## 4. Discussion

We used three scoring systems to predict different aspects of Taiwanese patients with AE. The APE score was used to predict the presence of neural-specific antibodies, and the results showed that it had high sensitivity (92.9%), low specificity (0%), and poor discrimination ability (AUC: 0.571). The RITE score was used to predict the response to immunotherapy, which also had high sensitivity (100%), low specificity (0%), and poor discrimination ability (AUC: 0.550). The NEOS score was used to predict the functional outcome at discharge and at 1 year or in long-term follow-up, and it showed low sensitivity, high specificity, and poor discrimination ability to predict the outcome at discharge but high sensitivity, high specificity, and good discrimination ability at 1 year or in long-term follow-up for all AE patients in our cohort. This suggests that the NEOS score may be a good prediction model for the long-term functional outcomes of AE patients.

In the original report, which used an APE score ≥ 4 to predict the presence of antibodies, the sensitivity was 82.6% and the specificity was 82% [7]. However, in our study, among the 21 patients who received the limbic encephalitis panel, the AUC of the APE score showed poor discrimination ability and low specificity. The reason for the low specificity in our study may be because the autoantibody test for AE was not covered by the National Health Insurance program in Taiwan, and therefore the patients without classical clinical features may have started immunotherapy without receiving the test. This emphasizes the fact that many patients with AE have ambiguous clinical symptoms and that objective biomarkers other than the currently available autoantibody tests are needed for diagnosis.

The RITE score was developed to evaluate the response to immunotherapy. In our cohort, the RITE score had poor discrimination ability to predict the response to immunotherapy. This may be because most of our patients had anti-NMDAR encephalitis, which usually has a good prognosis when treatment is initiated promptly. A previous study regarding anti-NMDAR encephalitis reported that 53% of their patients improved clinically after the first 4 weeks of treatment and that the other 47% may have had better outcomes if they had received further second-line treatment [11]. In most of our patients, the treatment started within 4 weeks of symptom onset, or they received early combined first-line treatment with second-line treatment (such as rituximab). This may have contributed to the good functional outcomes, as evidence suggests that the early use of first- and second-line immunotherapies can block the progression of neuronal damage [12]. Recently, one comprehensive literature review on immunotherapy in anti-NMDAR encephalitis also supported our point of early use of second-line treatment [6]. This review establishes a clear role for early immunotherapy and timely escalation to second-line treatment, especially with rituximab.

The NEOS score was initially designed to predict the functional outcomes of patients with anti-NMDAR encephalitis; however, its performance was not optimal in our study. We further applied the NEOS score to all our patients with AE and found that it had better discrimination ability for good functional outcomes at 1 year or in long-term follow-up. In the original NEOS study, the author reported that a higher NEOS score was strongly associated with the probability of poor functional status in patients with anti-NMDAR encephalitis [9]. Our results showed that the NEOS score had good predictive ability for long-term outcomes in patients with AE, regardless of the type.

Our results also showed no significant differences in clinical presentations between the survivor and non-survivor groups except for psychiatric symptoms and CSF protein elevation. The psychiatric symptoms were more prominent in the survivor group, and CSF protein elevation was more prominent in the non-survivor group. An elevated CSF protein level has been associated with the 1 year functional outcomes (mRS ≤ 2) of patients with AE [9], but the CSF protein level is not included in the NEOS score. Although some studies have suggested that ICU admission may be a prognostic factor [11,12], there was no significant difference in ICU admission between the two groups in our study.

Anti-NMDAR encephalitis has rarely been reported during pregnancy [13,14,15], and the diagnosis and treatment are challenging due to concerns over the safety of the mother and fetus. Pregnancy-related SE caused by AE is also rare [16,17]. The association between pregnancy and anti-NMDAR encephalitis is still unknown, but a pregnancy-related immune pathogenetic mechanism may be possible [18,19]. In our cohort, one patient was diagnosed with AE during pregnancy and improved clinically after a Cesarean section even without immunotherapy. Other case reports have also shown that clinical improvement may be related to delivery or that delivery seemed to accelerate recovery [13,14,15].

For teratoma treatment, previous studies have reported that removal of the teratoma may enhance the effectiveness and speed of action of the first-line treatment [20] and that patients with ovarian tumor removal may have better outcomes. However, some studies have reported an association between the presence of underlying tumors in AE patients and poor functional outcomes, seizures, and mortality [21,22]. In our cohort, two anti-NMDAR encephalitis patients with teratoma underwent teratoma removal surgery in the very early phase after diagnosis (within 2 weeks after first-line treatment); however, both still had a prolonged and complicated treatment course with a longer recovery period. We suggest that patients with a diagnosis of anti-NMDAR encephalitis and teratoma may need more aggressive and combined treatment and early removal of the teratoma, but that they still have to face a long duration of treatment and recovery.

Our results provide the first glimpse of how the three scoring systems worked in Taiwanese populations. APE and RITE performed suboptimally among our patient cohort. It is unclear why these two scores performed less well in this study, and it could be limited access to antibody testing or the differences in providing immunotherapy to AE. We only tested six commercially available antibodies; it could be that some of the high APE scores in patients are caused by unidentified antibodies. Most of our patients received immunotherapy within four weeks after AE onset. An earlier use of immunotherapy may contribute to a better outcome [6], and this might affect the outcome prediction of RITE. We also found that the NEOS score may be a good prediction score to assess long-term follow-up outcomes in all types of AE patients, not just patients with anti-NMDAR encephalitis. This may broaden their clinical utility. The limitations of this study include a relatively small sample size; however, our study is currently the largest validation study in Taiwan. A future study with more patients could verify the performance of these scores. In addition, another limitation of this present study was its retrospective design, so controlling for confounding factors was difficult. Furthermore, this study only included Taiwanese patients; the original scores included Americans in APE and RITE and Americans and Spanish in NEOS. This was an exploratory study, and future validation of our findings will still be needed.

## 5. Conclusions

APE and RITE scores had low AUCs in our cohort, suggesting a low prediction value for Taiwanese patients with AE. The NEOS score had a higher AUC for predicting functional outcomes at 1 year/long-term follow-up than at discharge. Accordingly, the NEOS score may be a good tool to predict functional outcomes over a longer follow-up period in Taiwanese patients, which may be suitable for all AE patients, not just those with anti-NMDAR encephalitis. The use of an optimal scoring system to predict severity or outcome may provide clinical physicians with clues on how to modify individual treatment strategies for AE patients.

## Figures and Tables

**Table 1:** Diagnostic criteria for possible autoimmune encephalitis.

1. A Rapid Progressive Course of Fewer than 3 Months of Working Memory Deficits, Altered Mental Status, or Psychiatric Symptoms
2. At least one of the following: New focal central nervous system findings; Seizures that cannot be explained by a previously known seizure disorder; CSF pleocytosis (white blood cell [WBC] count > five 5 cells per mm^3^); Magnetic resonance imaging (MRI) features suggestive of encephalitis;
3. Reasonable exclusion of alternative causes.

**Table 2:** Demographic data of Autoimmune encephalitis patients.

Characteristics	Number of Patients (27) and Percentage (100%)
Onset age, years (range)	26 (21–34.5)
Female N (%)	21 (78%)
Type of AE	
Anti-NMDAR encephalitis	14 (52%)
Other autoimmune antibodies	
Anti-ENA Ab	5 (18.5%)
Anti-thyroglobulin Ab	5 (18.5%)
Anti-TPO Ab	4 (14.8%)
Anti-SSA Ab	3 (11.1%)
ANA	1 (3.7%)
Anti-dsDNA Ab	1 (3.7%)
APE score ≥ 4	26 (96.3%)
RITE score ≥ 7	23 (95.8%)
NEOS score ≥ 4	2 (7.4%)
Onset symptom	
Consciousness disturbance	8 (29.6%)
Seizure	5 (18.5%)
Psychiatric symptoms	5 (18.5%)
Viral prodrome	4 (14.8%)
Fever	2 (7.4%)
Speech disturbance	2 (7.4%)
Memory decline	1 (3.7%)
Status epilepticus	20 (74.1%)
MRI (T2/FLAIR hyperintensity)	
Normal	6 (22.2%)
One or both medial temporal lobes	16 (59.2%)
Multifocal in gray matter, white matter	5 (18.5%)
Required general anesthesia for SE control	18 (66.7%)
Duration of hospitalization, days (range)	47 (31.5–69.5)
Admission to ICU	26 (96.3%)
Duration of ICU stay, days (range)	35 (15.5–55)
Treatment	
Steroid pulse	24 (88.9%)
IVIG	3 (11.1%)
Plasmapheresis/plasma exchange	9 (33.3%)
Plasmapheresis and plasma exchange three cycles	1 (3.7%)
Plasmapheresis four cycles	1 (3.7%)
Rituximab	7 (25.9%)
Cyclophosphamide	4 (14.8%)
Outcome	
Death during admission	5
Good outcome at discharge (mRS < 3)	19
Good outcome at long-term follow-up (mRS < 3)	20

Continuous variables were presented as medians (interquartile ranges). Categorical variables were presented as N (%). Abbreviations: AE = Autoimmune encephalitis; Anti-NMDAR encephalitis = Anti-N-methyl-D-aspartate receptor encephalitis; ANA = antinuclear antibody; Anti-ENA Ab = anti-Extractable Nuclear Antigen Antibody; Anti-TPO Ab = Anti-Thyroid Peroxidase antibody; Anti-SSA = anti–Sjögren’s-syndrome-related antigen A autoantibodies; Anti-dsDNA Ab = Anti-double stranded DNA antibodies; APE = Antibody Prevalence in Epilepsy score; RITE = Response to Immunotherapy in Epilepsy score; NEOS = anti-NMDAR Encephalitis One Year Functional Status score; SE = Status epilepticus; ICU = intensive care unit; IVIG = Immunoglobulin; and mRS = modified Rankin Scale.

**Table 3:** Clinical manifestations of patients with autoimmune encephalitis in the survivor and non-survivor groups.

	Survivor (*n* = 22)	Non-Survivor (*n* = 5)	*p*-Value
Gender			
Female	16	5	0.5552
Male	6	0	
Tumor			1
Ovarian teratoma	4	0	
Other malignancy	1	1	
Other autoimmune diseases			
Other autoimmune Ab	4	1	1
Only anti-thyroid Ab	3	1	1
Anti-thyroid Ab and other autoimmune Ab	1	0	0.63
Major symptoms			
Psychiatric symptoms	16	1	0.0473
Involuntary movement	9	0	0.1358
Speech disturbance	8	0	0.279
Viral prodrome	10	3	0.6483
Status epilepticus	16	4	1
Admission to ICU	21	5	1
Abnormal MRI finding	17	4	1
CSF finding			
Cell count ≥ 5	12	2	1
Protein ≥ 50 mg/dL	4	5	0.0016
IgG index > 0.65	11	3	
IgG index < 0.65	9	1	
No IgG index data	2	1	

Abbreviations; Ab = antibody; ICU = intensive care unit; and CSF = cerebrospinal fluid.

## Data Availability

The data presented in this study are available on request from the corresponding author. The data are not publicly available due to the patient privacy.
